# Pseudo superior vena cava entrance block during sinus rhythm uncovered by continuous atrial pacing

**DOI:** 10.1002/joa3.12740

**Published:** 2022-05-27

**Authors:** Takayuki Sekihara, Daisetsu Aoyama, Tomoya Eguchi, Hiroyasu Uzui, Hiroshi Tada

**Affiliations:** ^1^ Department of Cardiovascular Medicine, Faculty of Medical Sciences University of Fukui Fukui Japan

**Keywords:** catheter ablation, electrocardiography, heart conduction system, sinoatrial node, vena cava, superior

## Abstract

Usually, superior vena cava (SVC) entrance block is confirmed when SVC potentials disappear during sinus rhythm. We present a case of pseudo SVC entrance block during sinus rhythm, which was uncovered by continuous atrial pacing.

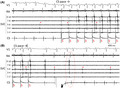

A 72‐year‐old man underwent radiofrequency (RF) catheter ablation including pulmonary vein isolation (PVI) and superior vena cava (SVC) isolation. After completing the PVI, we proceeded with empiric SVC isolation because he had a long SVC sleeve.^1^, ^2^ An activation map during sinus rhythm acquired before SVC isolation presented an intrinsic conduction block at the anterolateral side of the right atrium (RA)‐SVC junction,^3^, ^4^ whereas the posterior side was conductible (Figure 1A–C). We aimed to isolate the SVC without encircling ablation and started the ablation from the septal side toward the posterolateral side during sinus rhythm, and the SVC entrance block was observed without RF applications at the anterolateral side (Figure 1D). However, the SVC potentials reappeared when we started pacing from the coronary sinus ostium (Figure 2A). Acute reconnection was unlikely because this phenomenon was observed only during coronary sinus pacing, and SVC potentials disappeared when coronary sinus pacing was stopped (Figure 2B). Because the spontaneous SVC firing was conducted to the RA, it appeared that the exit block had also not been established at that point. Re‐mapping of the RA‐SVC junction during coronary sinus pacing revealed the RA‐SVC connection via the anterolateral side of the SVC that was not ablated **(**Figure 3 **)**. Under coronary sinus pacing, SVC potentials disappeared by adding RF application at the anterolateral side (Figure 4A,B). Circumferential ablation covering the whole anterolateral side was finally performed (Figure 4C) to eliminate the reconnection that occurred after 200 mcg/hr of isoproterenol infusion. We finally confirmed no dormant conduction upon 20 mg of adenosine triphosphate administration.

**FIGURE 1:**
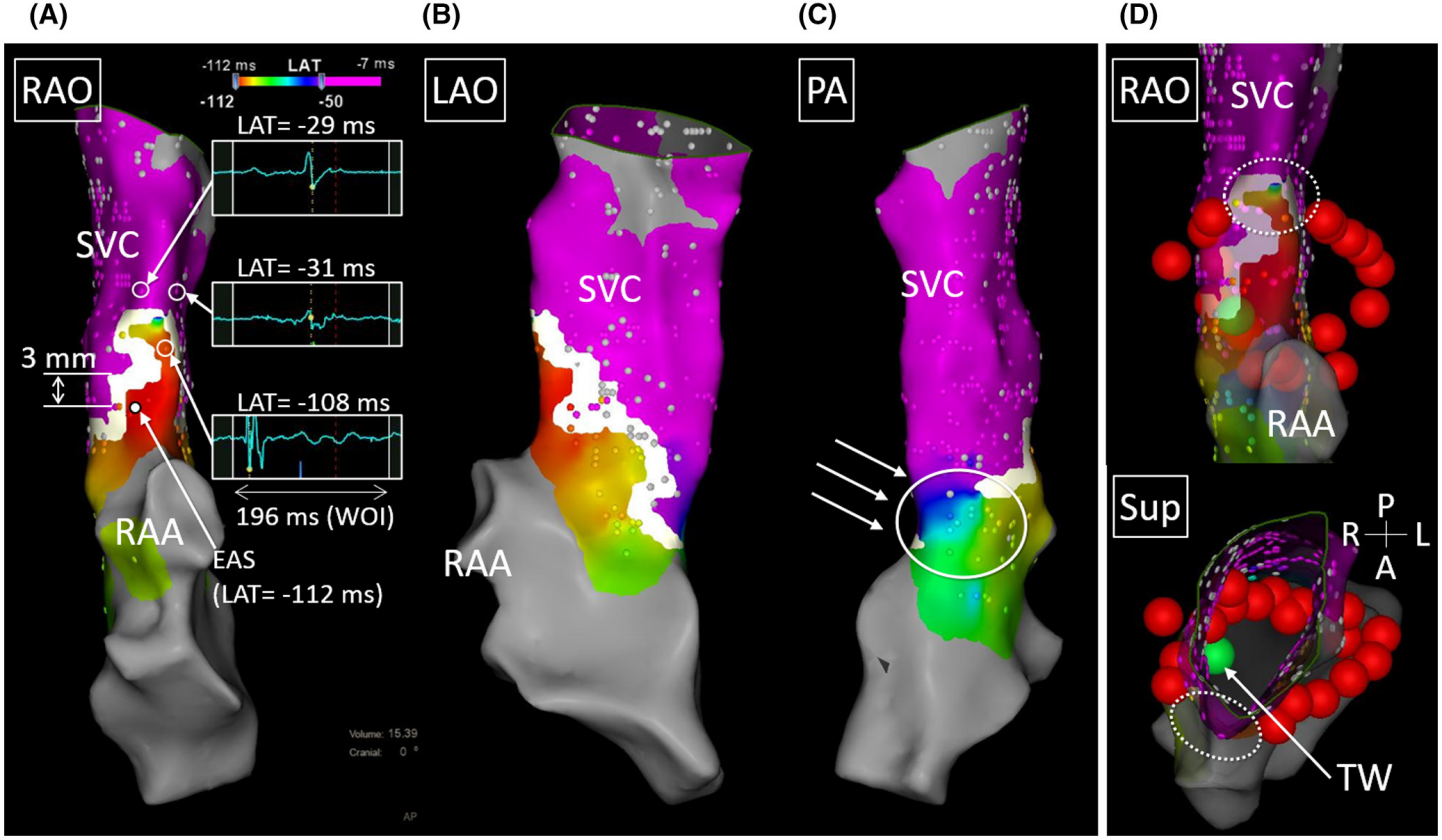
An activation map during sinus rhythm before starting SVC isolation. White lines at the RA‐SVC junction were generated by the early‐meets‐late function set at 29% (equals >30.4 ms splitting of the EGMs on the map). The anterolateral side of the RA‐SVC junction was functionally blocked (A). Solid arrows and circle indicate the conductible area at the posterior side (C). (D) Ablation points until achieving SVC entrance block during sinus rhythm superimposed on the activation map. Dotted circles indicate the anterolateral area not ablated. A, anterior; EAS, earliest activation site; L, left; LAO, left anterior oblique; LAT, local activation time; P, posterior; PA, posteroanterior; R, right; RAA, right atrial appendage; RAO, right anterior oblique; sup, superior; SVC, superior vena cava; TW, twitching site; WOI, window of interest.

**FIGURE 2:**
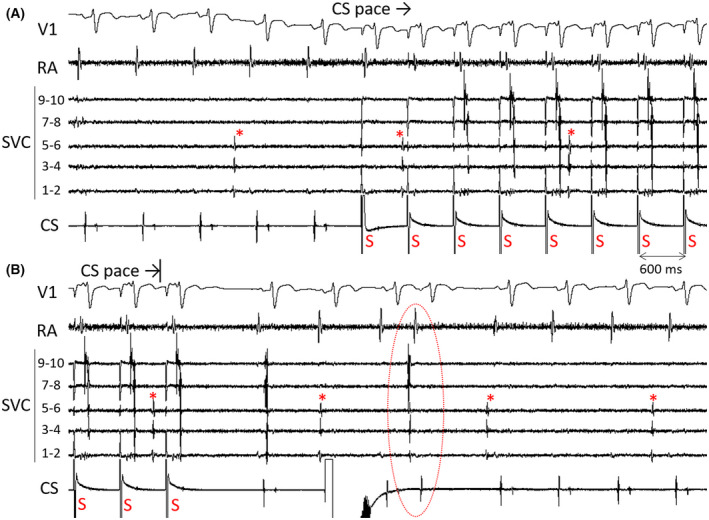
(A) After confirming the entrance block during sinus rhythm (80/min), SVC potentials reappeared during continuous coronary sinus pacing (100/min) following two non‐conductive beats. (B) Disappearance of the SVC potentials after stopping coronary sinus pacing. A dotted circle indicates a spontaneous SVC depolarization conducted to the RA. Asterisks indicate far‐field dissociated activities inside the right pulmonary vein. CS, coronary sinus; RA, right atrium; S, stimulation. Other abbreviations as in Figure 1.

**FIGURE 3:**
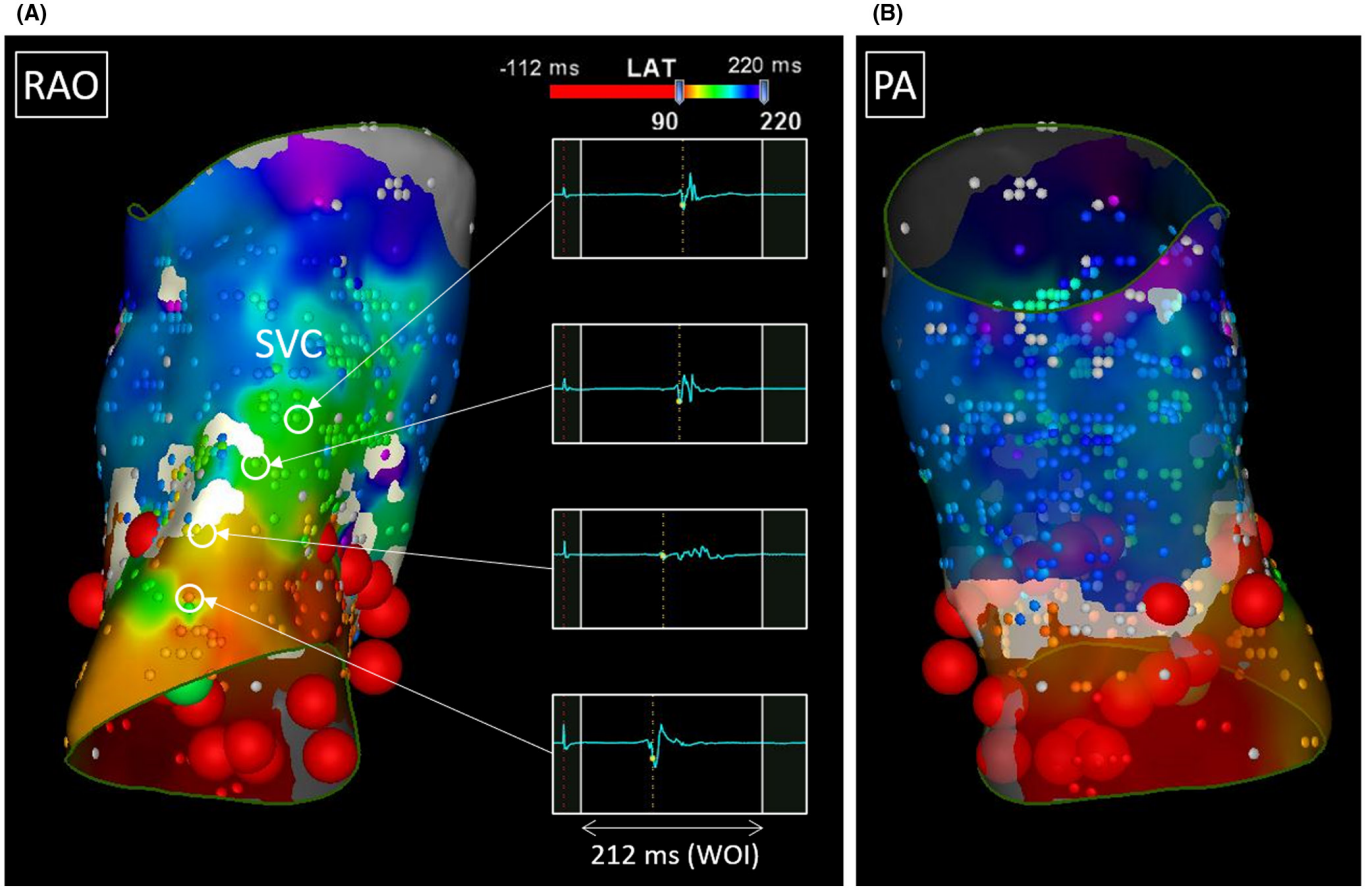
Re‐mapped activation map and local electrograms of the RA‐SVC junction. Ablation points until achieving the SVC entrance block during sinus rhythm were superimposed as in Figure 1D. White lines at the RA‐SVC junction were generated by the early‐meets‐late function set at 29% (equals >40.6 ms splitting of the EGMs on the map). Abbreviations as in Figure 1.

**FIGURE 4:**
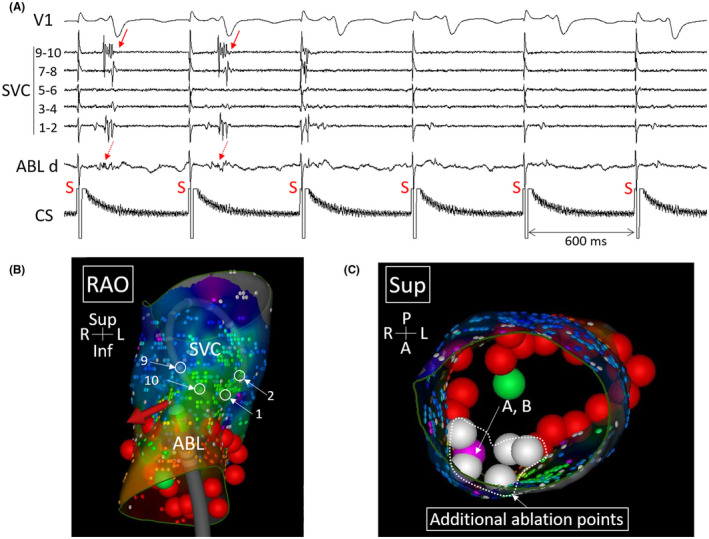
(A) Disappearance of superior vena cava (SVC) potentials (arrows) immediately after starting RF application (1 sec) at the anterolateral side of the SVC. (B) Catheter position during the RF application shown in (A) superimposed on the re‐mapped activation map shown in Figure 3. The electrode numbers of ring catheter correspond to those presented in (A). (C) Additional ablation points superimposed on the re‐mapped activation map (some additional ablation sites after isoproterenol infusion were missing). ABL, ablation catheter; d, distal. Other abbreviations as in Figures 1 and 2.

This case indicated that a careful investigation should be performed to confirm the SVC entrance block. Recently, a novel method for SVC isolation utilizing intrinsic conduction block area has been conceived.^3^ By using the method, the SVC entrance block during sinus rhythm was achieved without the ablation of the anterolateral side of the SVC (Figure 1D), which was close to the sinus node. A disappearance of SVC potentials during sinus rhythm is usually enough to confirm the SVC entrance block. However, SVC potentials reappeared when starting continuous coronary sinus pacing in this case. Because the SVC potentials disappeared when stopping the pacing, time‐dependent reconnection was unlikely. The conduction via the PV or left atrium was also unlikely because the right pulmonary vein was already isolated at that point, and the RA‐SVC conduction site was far from the left atrium.

These results indicated that the intrinsic conduction block area of the RA‐SVC junction could present persistent conduction by atrial pacing even after achieving the SVC entrance block during sinus rhythm. Therefore, the SVC entrance block during sinus rhythm might not be enough to confirm the true SVC entrance block. The sinus node has been reported to be located on the epicardial side of the terminal crest and insulated by the transitional cells, and sinus impulse exits from the node via the multiple preferential pathways.^5^ Probably, the preferential pathway conduction toward the SVC was intrinsically weak, or the pathway was further damaged by the RF deliveries. As a result, the entrance block during sinus rhythm was established even under residual RA‐SVC conduction during coronary sinus pacing. Another possible hypothesis is a bradycardia‐dependent conduction block (phase‐4 block). Diastolic depolarization of the sinus node or surrounding tissues might have caused a phase‐4 block.

This case illustrated a risk of misdiagnosing with superior vena cava entrance block when omitting RF applications at the intrinsic conduction block area close to the sinus node. Confirming the SVC entrance block both during sinus rhythm and continuous atrial pacing away from the sinus node might be necessary in the situation.

## CONFLICT OF INTEREST

Authors declare no conflict of interests for this article.

## PATIENT CONSENT STATEMENT

N/A (patient's clinical information was utilized based on the IRB approval #20180040).

## References

[1] Higuchi K , Yamauchi Y , Hirao K , Sasaki T , Hachiya H , Sekiguchi Y , et al. Superior vena cava as initiator of atrial fibrillation: factors related to its arrhythmogenicity. Heart Rhythm. 2010;7(9):1186–91. 20470902 10.1016/j.hrthm.2010.05.017

[2] Nogami A , Kurita T , Abe H , Ando K , Ishikawa T , Imai K , et al. JCS/JHRS 2019 guideline on non‐pharmacotherapy of cardiac arrhythmias. J Arrhythmia. 2021;37(4):709–870. 10.1002/joa3.12491 PMC8339126 34386109

[3] Tanaka Y , Takahashi A , Takagi T , Nakajima J , Takagi K , Hikita H , et al. Novel ablation strategy for isolating the superior vena cava using ultra high‐resolution mapping. Circ J. 2018;82(8):2007–15. 29877198 10.1253/circj.CJ-17-1352

[4] Inagaki D , Fukamizu S , Tokioka S , Kimura T , Takahashi M , Kitamura T , et al. A novel approach for effective superior vena cava isolation using the CARTO electroanatomical mapping system. J Arrhythmia. 2021;37(5):1295–302. 10.1002/joa3.12615 PMC8485816 34621428

[5] Fedorov VV , Schuessler RB , Hemphill M , Ambrosi CM , Chang R , Voloshina AS , et al. Structural and functional evidence for discrete exit pathways that connect the canine sinoatrial node and atria. Circ Res. 2009;104(7):915–23. 19246679 10.1161/CIRCRESAHA.108.193193 PMC2740656

